# Adverse drug reactions in drug information databases: does presentation affect interpretation?

**DOI:** 10.5195/jmla.2020.748

**Published:** 2020-01-01

**Authors:** Sean M. McConachie, Christopher A. Giuliano, Insaf Mohammad, Pramodini B. Kale-Pradhan

**Affiliations:** Eugene Applebaum College of Pharmacy and Health Sciences, Wayne State University, Detroit, MI, and, Beaumont Hospital, Dearborn, MI, et6398@wayne.edu; Eugene Applebaum College of Pharmacy and Health Sciences, Wayne State University, Detroit, MI, and and, Ascension St. John Hospital, Dearborn, MI, ek2397@wayne.edu; Eugene Applebaum College of Pharmacy and Health Sciences, Wayne State University, Detroit, MI, and and, Beaumont Hospital, Dearborn, MI, insaf@wayne.edu; Eugene Applebaum College of Pharmacy and Health Sciences, Wayne State University, Detroit, MI, and and, Ascension St. John Hospital, Dearborn, MI, pkale@wayne.edu

## Abstract

**Objective:**

Formatting of adverse drug reaction (ADR) information differs among drug information (DI) resources and may impact clinical decision-making. The objective of this study was to determine whether ADR formatting impacts adverse event interpretation by pharmacy practitioners and students.

**Methods:**

Participants were randomized to receive ADR information in a comparative quantitative (CQUANT), noncomparative quantitative (NQUANT), or noncomparative qualitative (NQUAL) format to interpret 3 clinical vignettes. Vignettes involved patients presenting with adverse events that varied in the extent to which they were associated with a medication. The primary outcome was interpretation of the likelihood of medication-induced adverse events on a 10-point Likert scale. Lower scoring on likelihood (i.e., ADR deemed unlikely) reflected more appropriate interpretation. Linear regression was performed to analyze the effects of ADR information format on the primary outcome.

**Results:**

A total of 108 participants completed the study (39 students and 69 pharmacists). Overall, the CQUANT group had the lowest average likelihood compared to NQUAL (4.0 versus 5.4; *p*<0.01) and NQUANT (4.0 versus 4.9; *p*=0.016) groups. There was no difference between NQUAL and NQUANT groups (5.4 versus 4.9; *p*=0.14). In the final model, at least 2 years of postgraduate training (−1.1; 95% CI: −1.8 to −0.3; *p*<0.01) and CQUANT formatting (−1.3; 95% CI: −0.9 to −1.7; *p*<0.01) were associated with reduced likelihood.

**Conclusions:**

Formatting impacts pharmacists’ and pharmacy students’ interpretation of ADR information. CQUANT formatting and at least two years of postgraduate training improved interpretation of adverse events.

## INTRODUCTION

Prescription drug utilization continues to rise in the United States. From 1999 to 2011, the prevalence of prescription drug use among adults increased from 51% to 59% and polypharmacy (i.e., 5 or more medications) increased from 8% to 15% [1]. Correspondingly, the prevalence of adverse drug reactions (ADRs) may also be increasing.

The US Food and Drug Administration reports that medication errors cause at least 1 death every day and injure approximately 1.3 million people annually [2]. Additionally, an estimated 5%–6% of all hospitalizations are due to ADRs, and over 15% of hospital admissions are complicated by an ADR [3, 4]. However, despite their high incidence and associated morbidity, ADRs can be difficult to identify in clinical practice. Most randomized controlled trials that lead to drug approval are not powered to detect rare adverse effects, which has led to a reliance on pharmacovigilance programs such as post-marketing surveillance, patient registries, and patient-level causality assessment to determine the likelihood of ADR occurrences in clinical practice [5–8]. Unfortunately, studies show that there is often little consensus among experts or assessment tools in identifying and classifying ADRs in real-world settings [9–12].

Pharmacists are frequently asked drug information (DI) questions regarding adverse events, and many pharmacists utilize DI databases to inform their clinical decisions [13, 14]. However, comparative evaluations of available DI resources demonstrate that there are major differences in the information presented in DI databases and their utility in answering DI questions [14–17]. Presentation of DI can impact interpretation of the risks and benefits of therapy and, therefore, clinical decision-making [18, 19]. Studies show that patients’ and clinicians’ perceptions of risk are influenced by the way drug risk and benefit information is displayed in patient handouts and clinical trial results [20–22]. For example, patient interpretation of risk and benefit information for a fictitious medication can be influenced by the presence or absence of corresponding placebo rates and quantitative differences in efficacy between placebo and medication [23]. Similarly, presentation of data in a quantitative versus qualitative format can also impact risk comprehension [23–25].

Ideally, DI databases should present information so that clinicians can quickly and effectively discriminate between adverse events that are more likely to be induced by medications (i.e., ADRs) as opposed to other etiologies. There are no studies evaluating the impact of ADR presentation in DI databases, nor are there studies that assess the interpretation of ADR information by pharmacists or pharmacy students. Therefore, the purpose of this study was to determine whether the presentation of ADR information in commonly used databases affects the interpretation of adverse events by pharmacists and pharmacy students. Additionally, the authors sought to determine whether differences in level of training impact interpretation of adverse events.

## METHODS

A randomized controlled trial was conducted at three medical centers (Ascension St. John Hospital, Detroit Medical Center, and Beaumont Hospital, Dearborn) and Wayne State University to evaluate the impact of presentation of ADR information on participants’ interpretation of adverse events. Pharmacists and fourth-year pharmacy students were recruited electronically via interdepartmental email lists and then randomized to receive three vignettes containing ADR information derived from one of three DI databases. This ADR information was used to evaluate three clinical vignettes in which a hospitalized patient was suspected of developing a specific ADR. Pharmacy residents were excluded as the researchers did not have access to an adequate number of potential respondents. Institutional review board approval was obtained prior to study commencement.

Prior to study commencement, a survey with three vignettes was developed (supplemental appendix). Each vignette presented respondents with a patient case involving a possible ADR. Respondents were asked to rate how likely they felt the suspected ADR was caused by medication on a 10-point Likert scale. All aspects of the case were identical except for the presentation of ADR information, which varied by group. In particular, information for the comparative quantitative formatting (CQUANT) group was derived from the Adverse Effects: In-Depth Answers section of the drug monograph in Micromedex, which included ADR incidence rates in a percentile format for both the medication and placebo and included other pertinent information related to the ADR. Although the In-Depth Answers section is not the default view for ADRs in Micromedex, this formatting was chosen to provide one group with a placebo-comparison frame, which is not available in the other two resources.

ADR information for the noncomparative quantitative formatting (NQUANT) group was derived from the Adverse Reactions section of the drug monograph in Lexicomp and included ADR incidence rates in percentile format for the medication without accompanying placebo rates. Finally, ADR information for the noncomparative qualitative formatting (NQUAL) group was derived from the Adverse Reactions section of the drug monograph in Epocrates Online and consisted of a list of ADRs categorized as “Serious” or “Common,” without accompanying incidence rates or placebo information. A breakdown of the differences between the ADR information formats is shown in Table 1.

**Table 1:** Summary of adverse drug reaction (ADR) information formats

	Noncomparative quantitative (NQUANT)	Comparative quantitative (CQUANT)	Noncomparative qualitative (NQUAL)
Drug database used for format and data abstraction	Lexicomp	Micromedex (In-Depth Answers)	Epocrates
Format of medication adverse drug reaction (ADR) incidence rates	Quantitative (percentage)	Quantitative (percentage)	Qualitative (e.g., “Common”)
Placebo comparison rates	Absent	Present (percentage)	Absent
Additional ADR information	Absent	Present (text)	Absent

The first two vignettes presented scenarios in which the adverse event was most likely due to the underlying disease state (i.e., ADR incidence was similar between placebo and medication or higher in the placebo group), and the last vignette presented a scenario in which the adverse event was more likely due to the suspected medication (i.e., ADR incidence was higher with medication compared to placebo). Likelihood was inverted for the last vignette. Overall, lower aggregate scores were representative of more appropriate interpretation across the 3 cases (i.e., ADR was deemed unlikely), whereas higher composite scores represented less appropriate interpretation. Content validation of the cases was performed by 4 pharmacists and 2 pharmacy residents. A raffle for 2 $50 gift cards was provided as an incentive for completion of the study. The vignettes and demographic questions were developed and administered through Qualtrics® (Provo, UT).

The primary outcome was interpretation of the likelihood of medication-induced adverse events averaged across the three vignettes. Secondary outcomes included differences in likelihood interpretation among respondents with lower or higher levels of training or experience with the DI resource. Training was stratified from lowest to highest level of training: doctor of pharmacy (PharmD) student, bachelor of pharmacy, PharmD, postgraduate year 1 (PGY1) residency, and postgraduate year 2 (PGY2) residency or fellowship. Additionally, demographic data were collected on the frequency of use of DI programs, whether participants had DI programs installed on a mobile device, how often participants believed their interventions related to ADRs were accepted by physicians in clinical practice, and total years of clinical experience.

Mean and standard deviation (SD), median and interquartile range, and n (%) were used to describe continuous, ordinal, and nominal data, respectively. Linear regression was performed to analyze differences between the independent variable of interest (ADR information format) and the dependent variable (confidence). Univariable analysis was performed using analysis of variance (ANOVA), Kruskal Wallis, or chi-square tests for continuous, ordinal, and categorical data, respectively. Post-hoc testing was done with Tukey honest significant difference (HSD) tests for ANOVA. Multivariable linear regression was performed to evaluate the effects of ADR formatting and level of training. Demographic variables were included in the multivariable analysis if there was an association with confidence and if there was a difference between the ADR formatting groups (*p*<0.1). Sample size was calculated to detect a medium effect size (*f*=0.25) for the intervention group and up to 6 additional independent variables. Using an alpha significance level of 0.05 and power of 80%, a total required sample size of 80 participants was calculated.

## RESULTS

One hundred and eighty-five participants started the study. Seventy-three responses were incomplete, and 4 participants were excluded because they were pharmacy residents. Therefore, 108 participants were included in the final analysis, consisting of 39 students and 69 pharmacists. Demographics of the 3 groups are displayed in Table 2.

**Table 2:** Baseline participant demographics

	NQUANT (n=40)		CQUANT (n=34)		NQUAL (n=34)	
Years of clinical experience	mean±standard deviation (SD)		mean±SD		mean±SD	

	6.8±9.0		6.4±7.7		7.4±8.7	
	n	(%)	n	(%)	n	(%)
	
Highest level of training						
Student	14	(35.0%)	12	(35.3%)	13	(38.2%)
Bachelor of pharmacy	5	(12.5%)	5	(14.7%)	5	(14.7%)
Doctor of pharmacy (PharmD)	9	(22.5%)	8	(23.5%)	3	(8.8%)
Postgraduate year 1 (PGY1)	7	(17.5%)	3	(8.8%)	11	(32.4%)
Postgraduate year 2 (PGY2) or fellow	5	(12.5%)	6	(17.6%)	2	(5.9%)
Prior use of assigned drug information (DI) resource	36	(90.0%)	25	(73.5%)	0	(−)
DI application installed on mobile device	31	(77.5%)	27	(79.4%)	25	(73.5%)

Most participants indicated they had used Micromedex and Lexicomp previously for DI, while none had used Epocrates. Most respondents (>60%) indicated they used DI resources to investigate ADRs in routine clinical practice at least once per day, with less than 15% of respondents indicating they used DI resources on a weekly basis or more sparingly. When utilizing DI resources, most participants (>50%) indicated that they consulted multiple resources to investigate ADRs either usually or always, and less than 10% indicated that they utilized multiple resources either rarely or never. Lastly, respondents estimated that their recommendations to clinicians regarding ADRs were accepted most of the time.

Overall, average likelihood was significantly different among the 3 groups (*f*(2,105)=10.52; *p*<0.01). Post-hoc Tukey HSD analyses revealed that average likelihood was lower for the CQUANT group than the NQUAL (mean difference −1.38 per case; *p*<0.01) or NQUANT (mean difference of −0.82 points per case; *p*=0.02) groups. There was no significant difference in likelihood interpretation between NQUAL and NQUANT groups (mean difference: 0.56 points higher in NQUAL group; *p*=0.14). These scores indicate that participants randomized to the CQUANT group more appropriately interpreted ADR data, on average, than respondents randomized to other groups.

**Figure 1:**
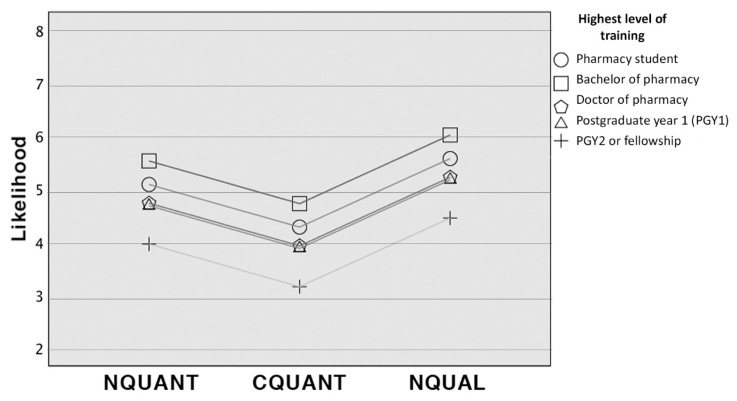
Likelihood stratified by highest level of training and ADR information format

There were no significant differences in highest level of training of respondents among groups; however, the highest level of training was significantly associated with likelihood (*f*(3,104)=3.39; *p*=0.01). Prior use of the assigned DI resource differed among groups (*χ*^2^(2)=66.4; *p*<0.05) but was not associated with likelihood interpretation (*t*(2)=−1.67; *p*=0.40). After adjusting for highest level of training, participants in the CQUANT group had a 1.3-point lower likelihood, on average, compared with those in the NQUAL group. Also, PGY2- trained or fellowship-trained pharmacists had a 1.1-point lower likelihood, on average, compared with students. The full regression model is displayed in Table 3, and the results are visualized in Figure 1.

**Table 3:** Effect of ADR information format and level of training on likelihood

	Unstandardized beta coefficient	95% Confidence interval	*p*-value
Constant	5.6		
NQUANT*	−0.5	−1.1 to 0.8	0.09
CQUANT*	−1.3	−1.9 to −0.7	<0.01
Bachelor of pharmacy†	0.5	−0.3 to 1.2	0.21
PharmD†	−0.3	−1.0 to 0.4	0.33
PGY1†	−0.4	−1.0 to 0.3	0.25
PGY2 or fellow†	−1.1	−1.8 to −0.3	<0.01

* Average difference in likelihood compared to NQUAL.

† Average difference in likelihood compared to pharmacy students.

## DISCUSSION

This study demonstrates that the presentation of ADR information in commonly used DI databases impacts interpretation by pharmacists and pharmacy students. The consequences of these findings could impact clinical practice. For example, pharmacists who deem that an ADR was more likely due to suboptimal formatting in DI resources could theoretically lead to inappropriate recommendations for medication discontinuation in instances where an adverse effect was more likely due to an underlying disease state. At worst, this could result in permanent discontinuation of a beneficial medication.

In our sample, over 68% of respondents indicated that their therapeutic recommendations were accepted either usually or all the time, demonstrating a path by which inappropriate adverse event interpretation could lead to poor patient care. Additionally, perceived ADRs from both providers and patients are a driving reason for why certain medications are not prescribed to patients who are likely to benefit from the therapy [26]. Observational studies of patients on statin therapy demonstrate that patients who experience intolerances to statins often permanently discontinue the therapy despite the fact that the majority of patients who are rechallenged are able to safely take the medication over the long term [27, 28]. It is possible that these misperceptions could be exacerbated by suboptimal ADR formatting in DI references.

In terms of differences between individual references, we found that CQUANT presentation of ADR formatting resulted in lower likelihood interpretation when ADRs were more likely to be due to the underlying disease state compared to formatting from other DI references. This finding likely stems from the fact that CQUANT (Micromedex In-Depth Answers) presented risk information for both placebo and medication in a comparative format and provided additional clinical trial information. This finding agrees with a previous study in which investigators randomized an online panel of 2,000 patients to receive fictitious medication efficacy information without placebo rates or in a comparative format with corresponding placebo event rates [23]. Participants who were presented with placebo incidence rates that were similar to those of the medication perceived lower efficacy for the medication than participants who were not given placebo information. This suggests that comparative placebo information impacts risk-benefit perception and, possibly, that placebo rates may be discounted if they are not included in DI references.

However, there was no evidence of a difference in likelihood interpretation between those randomized to receive information from NQUAL (Epocrates) and NQUANT (Lexicomp), both of which displayed ADR information without accompanying placebo rates, although the NQUAL group received ADR information in a qualitative format, whereas the NQUANT group received information in a quantitative format. This difference did not translate into a statistically significant difference in likelihood interpretation. None of the participants in the NQUAL group had used the Epocrates reference at baseline; however, we did not find that previous use of DI databases was associated with likelihood interpretation. This suggests that increased experience with a DI database does not improve ADR interpretation. Many respondents indicated that they utilized multiple resources for ADR interpretation in practice. Although widely considered a good practice, the results suggest that referencing multiple databases may be more beneficial if one of the databases frames risk information in a placebo-comparison format.

Our study also identified level of training as an independent predictor of likelihood interpretation. In particular, individuals with PGY2 residency or fellowship training interpreted ADRs as being less likely compared to students. However, there was no difference between PGY1-trained pharmacists or non-residency-trained pharmacists and students. The lack of difference between pharmacists with PGY1 training and students is surprising because current American Society of Health-System Pharmacists standards emphasize the understanding and detection of adverse drug events as a required objective in PGY1 residencies [29]. Additionally, a recent national survey suggested that over half of PGY1 pharmacy practice residencies incorporated longitudinal DI projects throughout residency, and many had formal DI rotations [30]. Colleges of pharmacy are also required to incorporate pharmacoepidemiology, health informatics, and health information evaluation into their curricula [31]. The differences in training that lead to more appropriate clinical interpretation of ADRs by PGY2-trained warrants further research to improve pharmacy-led patient safety initiatives; however, in the interim, our findings suggest that optimizing ADR presentation can help as well.

Our study has a number of limitations. First, the formatting options might not be truly representative of ADR information in all DI resources, as we only used three available drug references. Additionally, the CQUANT formatting was drawn from the In-Depth Answers section of the Micromedex drug monograph, which was not the default view for ADR information. It was unclear how often clinicians used this information due to time constraints in clinical practice or if they were even aware of this resource. Thus, our results do not suggest that Micromedex, in general, is a superior reference for interpreting ADRs, but they do suggest that the formatting found in the In-Depth Answers section is superior to other methods of formatting.

There is also subjectivity in adverse event interpretation in clinical practice at baseline. For example, past experiences with a particular medication could likely affect interpretation of ADR information, which could potentially confound the results. Also, our sample population only included pharmacy students from a single college of pharmacy, and pharmacists were polled from three health systems. We also excluded key clinician groups such as physicians, mid-level providers, and pharmacy residents. These restrictions might limit the external validity of our findings.

Finally, our clinical vignettes lacked the breadth of information available to clinicians in a real-life setting where additional clinical resources, electronic medical record information, information obtained from patient interviews, and collaboration with the health care team could yield further information on temporality, previous exposures, and alternative causes, factors that are important in the assessment of causality for potential ADRs [32].

Despite these limitations, our study demonstrates that the formatting of DI references impacts the interpretation of ADR information by pharmacists and pharmacy students. Respondents who received ADR information that was formatted to include concomitant placebo rates and respondents with PGY2 residency or fellowship experience deemed the potential ADRs to be less likely and more appropriately interpreted clinical vignettes. Further studies are needed to determine the impact of DI database formatting on other types of health care professionals and to identify optimal methods for educating health care professionals and students on their appropriate use. In the future, addition of placebo event rates may improve clinical decision-making among users of DI databases.

## SUPPLEMENTAL FILE

Appendix Survey and clinical vignettes

## 

**Sean M. McConachie**, et6398@wayne.edu, Eugene Applebaum College of Pharmacy and Health Sciences, Wayne State University, Detroit, MI, and, Beaumont Hospital, Dearborn, MI

**Christopher A. Giuliano**, ek2397@wayne.edu, Eugene Applebaum College of Pharmacy and Health Sciences, Wayne State University, Detroit, MI, and and, Ascension St. John Hospital, Dearborn, MI

**Insaf Mohammad**, insaf@wayne.edu, Eugene Applebaum College of Pharmacy and Health Sciences, Wayne State University, Detroit, MI, and and, Beaumont Hospital, Dearborn, MI

**Pramodini B. Kale-Pradhan**, pkale@wayne.edu, Eugene Applebaum College of Pharmacy and Health Sciences, Wayne State University, Detroit, MI, and and, Ascension St. John Hospital, Dearborn, MI
