# Supplementary material for: Adverse drug reactions in drug information databases: does presentation affect interpretation?
Source: J Med Libr Assoc. 2020 Jan 1;108(1):76–83. doi: 10.5195/jmla.2020.748 (PMC6919994; doi:10.5195/jmla.2020.748)
Supplement: Appendix [file jmla-108-76-s001.pdf]

JMLA Journal of the Medical Library Association

SC

Supplemental content to
J Med Libr Assoc. Jan;108(1):dx.doi.org/10.5195/jmla.2020.748
www.jmla.mlanet.org
© McConachie, Giuliano, Mohammad, Kale-Pradhan 2020

Adverse drug reactions in drug information databases: does presentation affect interpretation?

Sean M. McConachie; Christopher A. Giuliano; Insaf Mohammad; Pramodini B. Kale-Pradhan

APPENDIX
Survey and clinical vignettes

1. Which of the following categories do you identify with most?
   a. Pharmacy student
   b. Pharmacy resident
   c. Clinical pharmacist
   d. Clinical pharmacist specialist
   e. Pharmacy manager

2. What is your highest level of pharmacy training?
   a. Doctor of pharmacy (PharmD) candidate
   b. Bachelor's of science (BS) in pharmacy (Pharm)
   c. PharmD
   d. postgraduate year 1 (PGY1) residency
   e. postgraduate year 2 (PGY2) residency
   f. Fellowship

3. Which of the following drug information databases do you use to evaluate adverse drug reactions? (Select all that apply)
   a. Lexicomp
   b. Micromedex
   c. Clinical Pharmacology
   d. Epocrates Online
   e. Rxlist.com
   f. Other, please explain

4. Which of the following drug information applications do you use on your cellular phone or portable device?
   a. Lexicomp
   b. Micromedex
   c. Epocrates
   d. Clinical Pharmacology
   e. Other, please explain

5. How often do you use electronic drug information databases for the purposes of investigating adverse drug reactions?
   a. Multiple times per day
   b. Daily
   c. Several times per week
   d. Weekly
   e. Monthly
   f. Never

JMLA Journal of the Medical Library Association

SC
Supplemental content to
J Med Libr Assoc. Jan;108(1):dx.doi.org/10.5195/jmla.2020.748
www.jmla.mlanet.org
© McConachie, Giuliano, Mohammad, Kale-Pradhan 2020

6. How often do you consult multiple drug information databases for a question regarding adverse drug reactions?
   a. Never
   b. Rarely
   c. Sometimes
   d. Usually
   e. Always

7. In your experience, how often do clinicians accept your recommendations regarding the modification of medication regimens secondary to expected adverse drug reactions?
   a. Never
   b. Rarely
   c. Sometimes
   d. Usually
   e. Always

8. You receive a call from a physician regarding an internal medicine patient with a past medical history of cirrhosis who is being treated for hepatic encephalopathy with lactulose and rifaximin. It is day 3 of therapy, and the medical team noted the patient has ascites. The medical team is wondering if it could be caused by rifaximin. The following ADR information is listed in a drug information reference:

Noncomparative quantitative (NQUANT) adverse drug reaction (ADR) information (derived from Lexicomp)

**Adverse Drug Reactions:**<br>Cardiovascular: Peripheral edema (15%)<br>Central nervous system: Dizziness (13%), fatigue (12%)<br>Hepatic: Ascites (11%)

Comparative quantitative (CQUANT) adverse drug reaction (ADR) information (derived from Micromedex)

**Adverse Drug Reactions (In-Depth): Ascites**<br>1) Incidence: 11%<br>2) Hepatic encephalopathy (oral route): 11% vs 9% with placebo

Noncomparative qualitative (NQUAL) adverse drug reaction (ADR) information (derived from Epocrates)

**Adverse Drug Reactions**<br><u>Serious Reactions</u><br>Superinfection, C. difficile-associated diarrhea, hypersensitivity reaction, angioedema, anaphylaxis, exfoliative dermatitis<br><u>Common Reactions</u><br>Peripheral edema, nausea, dizziness, fatigue, ascites, headache, muscle spasms, pruritus, abdominal pain, anemia, depression, nasopharyngitis, arthralgia, dyspnea, fever, rash, ALT increased

On a scale of 1–10, how confident are you that this reaction was caused by rifaximin based on the information provided? (1=Very unlikely, 10=Very likely)

JMLA Journal of the Medical Library Association

SC

Supplemental content to
J Med Libr Assoc. Jan;108(1):dx.doi.org/10.5195/jmla.2020.748
www.jmla.mlanet.org
© McConachie, Giuliano, Mohammad, Kale-Pradhan 2020

9. You receive a call from a physician regarding an internal medicine patient with chronic pancreatitis who is receiving pancrelipase with meals. It is day 2 of therapy, and the medical team noted the patient has continued abdominal pain. The medical team is wondering if it could be caused by pancrelipase. The following ADR information is listed in a drug information reference:

NQUANT ADR information (derived from Lexicomp)

Adverse Drug Reactions:
>10%:
Central nervous system: Headache (3% to 15%)
Gastrointestinal: Abdominal pain (3% to 18%)
Hematologic & oncologic: Lymphadenopathy (11%)
1% to 10%:
Gastrointestinal: Dyspepsia (10%), diarrhea (≤10%), flatulence (3% to 9%), choledocholithiasis (7%), early satiety (6%), vomiting (6%), upper abdominal pain (≤5%), abnormal stools (≤4%)

CQUANT ADR information (derived from Micromedex)

Adverse Drug Reactions (In-Depth): Abdominal Pain
1) Summary
a) Abdominal pain was one of the most common treatment emergent adverse events reported during clinical trials; however, in all studies the incidence rate was lower in the pancrelipase group compared with the placebo group.
2) Incidence: 9% to 18%
3) In a clinical trial evaluating cystic fibrosis patients (8 to 57 years of age) with exocrine pancreatic insufficiency, abdominal pain was reported in 10% of patients who received pancrelipase (dose of 6300 lipase units/kg/day; n=20) compared with 15% of patients who received placebo (n=20) over 8 to 26 days. Upper abdominal pain was also reported more frequently in placebo-treated patients (5% vs 15%, respectively).
4) Abdominal pain has been commonly reported with both delayed- and immediate-release pancreatic enzyme products containing pancrelipase for the treatment of exocrine pancreatic insufficiency. In a randomized, double-blind, crossover study in cystic fibrosis patients with exocrine pancreatic insufficiency (n=32), abdominal pain occurred less frequently with pancrelipase (dose of 4000 lipase units per gram of fat ingested per day) than with placebo (n=31) (9% vs 26%), respectively.
5) In a randomized, double-blind, placebo-controlled, 2-treatment, crossover study of patients (age 7 to 23 years), abdominal pain was reported less frequently in patients with exocrine pancreatic insufficiency due to cystic fibrosis who received pancrelipase treatment, 18% (6/34) compared with 28% (9/32) of patients who received matching placebo treatment. The incidence was similar in children (7 to 11 years), adolescents (12 to 16 years), and adults (greater than 18 years). The mean exposure to pancrelipase was 30 days. Patients were randomized to receive either titrated doses not greater than 2500 lipase units/kg/meal of pancrelipase or matching placebo for 6 to 7 days, followed by crossover treatment for another 6 to 7 days.
6) Abdominal pain was one of the most commonly reported gastrointestinal adverse events in an open-label, uncontrolled study of 19 patients (1 to 6 years old) with exocrine pancreatic insufficiency due to cystic fibrosis who received pancrelipase treatment at titrated doses (dose no greater than 2500 lipase units/kg per meal) for 14 days.

JMLA Journal of the Medical Library Association

SC

Supplemental content to
J Med Libr Assoc. Jan;108(1):dx.doi.org/10.5195/jmla.2020.748
www.jmla.mlanet.org
© McConachie, Giuliano, Mohammad, Kale-Pradhan 2020

NQUAL ADR information (derived from Epocrates)

Adverse Drug Reactions
Serious Reactions
Hypersensitivity reaction, fibrosing colonopathy, intestinal obstruction, viral transmission risk
Common Reactions
Abdominal pain, headache, diarrhea, flatulence, constipation, abnormal stools, nausea/vomiting, irritability, appetite decreased, pruritus, urticarial, rash, cough, dizziness, early satiety, weight loss, dyspepsia, pharyngolaryngeal pain, hyperuricemia, hyperglycemia, hypoglycemia, biliary tract stones

On a scale of 1–10, how confident are you that this reaction was caused by pancrelipase based on the information provided? (1=Very unlikely, 10=Very likely)

10. You receive a call from a physician regarding an internal medicine patient with pulmonary arterial hypertension who is being treated with ambrisentan at home. The patient presents with a lower hemoglobin than his previous baseline. The medical team is wondering if it could be caused by ambrisentan. The following ADR information is listed in a drug information reference:

NQUANT ADR information (derived from Lexicomp)

Adverse Drug Reactions
Cardiovascular: Peripheral edema (14% to 38%), flushing (4%)
Central nervous system: Headache (3%)
Gastrointestinal: Dyspepsia (3%)
Hematologic and oncologic: Decreased hemoglobin (7% to 10%; dose-dependent), anemia (7%), decreased hematocrit

CQUANT ADR information (derived from Micromedex)

Adverse Drug Reactions (In-Depth): Decreased Hemoglobin
a.) Incidence: 7% to 10%
b.) Decreases in hemoglobin concentration and hematocrit, sometimes greater than 15% from baseline or persisting for up to 4 years of treatment, have occurred with administration of ambrisentan. The mean decrease in hemoglobin from baseline to treatment discontinuation in one 12-week placebo-controlled trial was 0.8 g/dL with ambrisentan therapy. Marked decreases in hemoglobin (greater than 15% decrease from baseline) occurred in 7% of ambrisentan-treated subjects (10% of patients receiving 10mg) compared with 4% of placebo-treated subjects. These decreases stabilized after the first few weeks of treatment and did not appear to result from hemorrhage or hemolysis. Mean decreases in hemoglobin concentrations ranging from 0.9 g/dL to 1.2 g/dL from baseline continued for up to 4 years of therapy in a long-term, open-label extension of 2 clinical studies. Decreases in hemoglobin and hematocrit requiring treatment by blood transfusion have also been reported during postmarketing surveillance. Consider treatment discontinuation if a clinically significant decrease in hemoglobin occurs and other causes have been excluded.

Journal of the Medical Library Association

Supplemental content to
J Med Libr Assoc. Jan;108(1):dx.doi.org/10.5195/jmla.2020.748
www.jmla.mlanet.org
© McConachie, Giuliano, Mohammad, Kale-Pradhan 2020

NQUAL ADR information (derived from Epocrates)

Adverse Drug Reactions:
Serious Reactions
Hypersensitivity reaction, fluid retention (severe), anemia
Common Reactions
Peripheral edema, hemoglobin decrease, nasal congestion, sinusitis, flushing, ALT/AST elevation, sperm count decrease

On a scale of 1–10, how confident are you that this reaction was caused by pancrelipase based on the information provided? (1=Very unlikely, 10=Very likely)

11. Approximately how many years of clinical experience do you have?
